# COVID-19 and tourism sector stock price in Spain: medium-term relationship through dynamic regression models

**DOI:** 10.1186/s40854-022-00402-0

**Published:** 2023-01-06

**Authors:** Isabel Carrillo-Hidalgo, Juan Ignacio Pulido-Fernández, José Luis Durán-Román, Jairo Casado-Montilla

**Affiliations:** 1 Laboratory of Analysis and Innovation in Tourism, University of Jaén, Paraje Las Lagunillas S/N, D3-134, 23071 Jaén, Spain; 2 Laboratory of Analysis and Innovation in Tourism, University of Jaén, Paraje Las Lagunillas S/N, D3-273, 23071 Jaén, Spain; 3 Laboratory of Analysis and Innovation in Tourism, University of Jaén, Paraje Las Lagunillas S/N, D3-217, 23071 Jaén, Spain

**Keywords:** COVID-19, Stock exchange, Tourism stock, Dynamic regression models, Spain

## Abstract

The global pandemic, coronavirus disease 2019 (COVID-19), has significantly affected tourism, especially in Spain, as it was among the first countries to be affected by the pandemic and is among the world’s biggest tourist destinations. Stock market values are responding to the evolution of the pandemic, especially in the case of tourist companies. Therefore, being able to quantify this relationship allows us to predict the effect of the pandemic on shares in the tourism sector, thereby improving the response to the crisis by policymakers and investors. Accordingly, a dynamic regression model was developed to predict the behavior of shares in the Spanish tourism sector according to the evolution of the COVID-19 pandemic in the medium term. It has been confirmed that both the number of deaths and cases are good predictors of abnormal stock prices in the tourism sector.

## Introduction

The global coronavirus disease 2019 (COVID-19) pandemic declared in March 2020 has blocked and weakened the global economy, with tourism being the most affected and distorted sector. To reduce the spread of infection, governments worldwide responded with a series of decisive and radical action policies to reduce people’s movements and interactions (Chai 2021; Liew 2020a).

The COVID-19 pandemic has had a devastating effect on the tourism sector, as health-related crises “influence the perception of tourism risk, causing a sudden decline in demand, with significant socio-economic repercussions, especially in countries dependent on tourism” (Novelli et al. 2018:85). Global international arrivals have fallen by 73% in 2020 and 71% in 2021 (UNWTO 2021).

Spain was among the first countries to experience high levels of COVID infections. Notably, tourism is among Spain’s main productive sectors, accounting for 12% of its gross domestic product (GDP), which saw its activity reduce to minimum levels during the pandemic (Gil-Alana and Poza 2022), with a 77% decline in international arrivals in 2020 and 63% in 2021. Accordingly, Spain was among the countries that registered a greater drop in the sector’s turnover (Skare and Riberio 2021), resulting in minimal employability data and a higher rate of unemployment (Chen 2020).

Regarding the stock market, no previous infectious disease outbreak has affected it as strongly as the COVID-19 pandemic (Baker et al. 2020). This pandemic interacts negatively with the tourism sector stock market (Al-Awadhi et al. 2020), experiencing a substantial decline in the valuation of tourism-related companies (Sharma and Nicolau 2020).

Spain is the world’s second-largest tourist destination, with 83.7 million international arrivals in 2019 (UNWTO 2020). Assuming the effect of COVID-19 on tourism and the economy of the country and following Kusumahadi and Permana (2021), who highlight the need to comprehensively examine the factors that affect stock price performance during COVID-19, the objectives of this study are as follows:

1. Ascertaining the negative effect of this pandemic on the tourism sector stock market.
2. Determining the evolution variables of COVID-19 that best predict the tourism stock price.
3. Developing a prediction model for the behavior of shares in Spain’s tourism sector according to the evolution of the COVID-19 pandemic.

Accordingly, we conduct a descriptive analysis, in which the behavior of the tourism sector stock market and Ibex 35^1^ are analyzed and compared. Additionally, we apply a dynamic regression model (DRM) with the tourism sector’s abnormal stock price (ASP) as a dependent variable on the number of deaths and positive cases of COVID-19.

Event studies (Chai 2021; Liew 2020a; Nhamo et al. 2020) and classic regression models (Carter et al. 2022; Gil-Alana and Poza 2022; Haroon and Rizvi 2020; Liew 2020b; Sharma and Nicolau 2020; Wu et al. 2021) are the most used methodologies in the studies conducted so far to demonstrate the effect of COVID-19 on the tourism sector stock market. Therefore, the development of prediction models using the DRM is novel.

The analysis presented here complements the existing scientific literature on the effect of COVID-19 on the tourism sector stock market, emphasizing specific aspects of the tourism industry, as most studies conducted to date analyze the stock market generally.

The main difference regarding existing studies is that a database spanning a long period of time (14 months) will be used to conduct a medium-term analysis,^2^ thereby filling the current gap in the scientific literature, as all related studies were conducted in the early stages of the pandemic, mostly ending in March 2020, as detailed in the literature review section. (Al-Awadhi et al. 2020; Carter et al. 2022; Huo and Qiu 2020; Liew 2020b; Nhamo et al. 2020; Sharma and Nicolau 2020). Sharma and Nicolau (2020) indicate that the decline in the stock price of tourism companies justifies concerns about the medium-term outlook, a period in which the sector's recovery is expected (Škare et al. 2021).

In the methodology used thus far by the scientific literature, only some authors have sought to link the number of positive cases with the behavior of the tourism sector stock market (Liew 2020b; Sharma and Nicolau 2020, Wu et al. 2021), and Sharma and Nicolau (2020) relate it to the number of deaths, without determining the better predictor. Therefore, another fundamental contribution of this study is determining the best explanatory variable (cumulative number of deaths or positive cases) that predicts tourism companies’ stock prices.

This study examines Spain, a tourism-oriented country, where no other studies of this kind have been conducted previously. Only Gil-Alana and Poza (2022) have analyzed the effect of COVID-19 on the Spanish tourist stock market but only in a short-term and descriptive manner.

Consequently, the research presented here confirms and quantifies the negative medium-term effect of COVID-19 on the tourism sector stock market, which is greater than its effect on the rest of the market. Furthermore, the cumulative numbers of cases and deaths caused by COVID-19 and the evolution of stock prices in the tourism sector during the pandemic are negatively related. Moreover, they are considered good predictors of the same.

Policymakers, researchers, and practitioners need frameworks and information that guide them in undertaking effective and good decisions (Estiri et al. 2022). The results presented here are useful and important because, in the case of a pandemic, there is a need to know not only the severity of the pandemic but also how damaging it is perceived to be by the market and investors (Ru et al. 2020). This is because their expectations will influence companies' stock prices (Nhamo et al. 2020). “The risk assessment of unexpected natural disasters can provide the basis for policymakers and tourism operators to make risk emergency strategies and tourism planning” (Chen et al. 2022:319).

This study demonstrates persistent effects during the COVID-19 period on the tourism sector stock market, which will help practitioners make decisions in the medium term. Therefore, managers and policymakers can radically modify the strategy expecting a persistent effect rather than just taking temporary measures to cushion the initial effect (Gil-Alana and Poza 2022), thus focusing appropriately on their aid and support (Gil-Alana and Poza 2022). Furthermore, this is beneficial information for investors, who should be aware of financial risks (Amin et al. 2021) when it comes to making decisions to maintain their wealth during pandemics, as it allows them to anticipate and protect their results by diversifying their portfolios (Sharma and Nicolau 2020). In addition to capital markets, stock market shocks damage economic development; therefore, being able to predict stock price crash risk can protect shareholder and investor values (Wen et al. 2019).

## Literature review

On March 11, 2020, the World Health Organization declared COVID-19 a global pandemic, one of the most serious in terms of the effects and number of deaths noted in the last century, resulting in major outbreaks worldwide. This pandemic has significantly affected the global economy, and its evolution is fraught with uncertainty (Ramelli and Wagner 2020).

Pandemic crises have lasting negative effects on the tourism industry and economy (Škare et al. 2021). Tourism has been among the most affected sectors by the COVID-19 pandemic (Bartik et al. 2020), owing to lockdowns and quarantine measures and the closure of borders, resulting in a decline of between 65 and 90% in international movements (UNWTO 2021).

In relation to the stock market, the negative effect of COVID-19 on stock prices is far greater than any previous outbreak of infectious diseases, such as SARS-2003, Spanish flu, or Ebola (Nhamo et al. 2020). Moreover, it is easy to access much more information quickly than at that time (Baker et al. 2020). The efficient market hypothesis (Malkiel 1989) explains this effect. “The region with more confirmed cases would suffer more substantial economic losses, the profitability of companies in that area would be weakened, and their stock returns would decrease” (Sun et al. 2021:1). Behavioral finance studies show that investors’ emotions and sentiments influence their investment decisions, thereby affecting stock market prices, returns, and volatility. Thus, the stock market correlates positively with negative (COVID-19-related terms) and positive (by COVID-19 vaccine-related terms) investor sentiment (Cevik et al. 2022). Wen et al. (2019) demonstrated a negative, direct, and significant relationship between stock price crash risk and retail investor attention.

Over time, the market-listed prices of tourism companies have been analyzed to understand the effect of extraordinary events such as terrorism and war (Chen 2011; Drakos 2004; Demiralay and Kilincarslan 2019; Kim and Gu 2004; Lanouar and Goaied 2019), and epidemic diseases (Chen et al. 2007; Ichev and Marinč 2018; Kim et al. (2020); Mckercher and Chon 2004; Novelli et al. 2018). The results obtained from these studies indicate that tourism is highly vulnerable and has a devastating effect with significant economic losses.

Ebola (Ichev and Marinč 2018) or other outbreaks of infectious and macroscopic epidemic diseases such as influenza A, swine flu, bovine spongiform, or salmonella (Kim et al. 2020) had a direct and negative impact on the tourism sector stock market and other related sectors above other industries.

Regarding the SARS-2003 pandemic outbreak, although there are more differences than similarities between SARS-19 and COVID-2019 (Chen et al. 2022; Hassan et al. 2020), it can be considered the predecessor to the current coronavirus. Abnormal stock returns in the tourism sector showed significantly negative cumulative abnormal yields or lower stock prices during the SARS-2003 outbreak (Chen et al. 2018). The SARS-2003 crisis affected tourism companies more negatively than other industries (Chen et al. 2007), displaying similar behavior to other epidemics, such as the bird flu (Chen et al. 2009). This demonstrates the fragility of the tourism sector in the face of an epidemic and implies that a new epidemic could send stock markets plummeting.

Regarding the effect of COVID-19 on the tourism sector and the stock market generally, following a comprehensive review of the literature, Table 1 summarizes the most important scientific literature, specifically the methodology used, geographical area, sector, and each research’s areas of focus in the stock market, and the time frame.

**Table 1:** Scientific literature analyzed on the relationship between COVID-19 and the stock market

	Methodology	Geographical scope	Stock market scope/sector	Time frame
Ahmed (2020)	Classic regression model	Pakistan	Stock indexes	01/2020–06/2020
Ahmed et al. (2021)	Welch test, HI t-test, Generalized Method of Moment	South Asian countries	Commodities(gold and oil) and stock indexes	03/06/2019–13/03/2020
Alomari et al. (2022)	Quantile regressions	USA	Stock indexes and multisectoral analysis (no tourism)	01/1985–03/2020
Amin et al. (2021)	Panel data regression	South America, North America and Central America	Stock indexes	10/03/2020–09/04/2020
Ashraf (2020)	Panel data regression	64 Countries (included Spain)	Stock indexes	22/01/2020–17/04/2020
Al-Awadhi et al. (2020)	Panel data regression	China	Stock indexes and multisectoral analysis, differentiating air transportation and hotels	10/01/2020–16/03/2020
Al-Qudah, and Houcine (2021)	Panel data regression and event study	Africa, Americas, Eastern Mediterranean, Europe, South-East Asia and Western Pacific	Stock indexes	21/01/2020–11/03/2020
Baek and Lee (2021)	Markov switching model and BEKK-multivariate GARCH model	USA	Stock indexes and multisectoral analysis (no tourism)	02/01/2020–30/04/2020
Baker et al. (2020)	Descriptive. Text-based methods	USA	Stock market	01/1985–06/2020
Bañuls (2021)	Descriptive analysis	USA, Europe, Spain and China	Stock indexes	19/02/2020–15/03/2020
Ben Amar et al. (2021)	Diebold and Yilmaz (2012) spillover index approach	Europe, North America, Latin America, Asia and Pacific, GCC countries	Stock indexes	31/12/2019–30/06/2020
Carter et al. (2022)	Multivariate regression model and event study	USA	Airlines, restaurants, and hotels	15/02/2020–30/03/2020
Chai (2021)	Event study (case study)	China	Stock index and tourist company (Caissa Tourism)	01/04/2019–08/01/2020
Ganie et al. (2022)	Event study	USA, India, Brazil, Mexico, Russia and Spain	Stock indexes	01/2020–09/2020
Gil-Alana and Poza (2022)	Fractional integration-regression model	Spain	Stock index and tourism	14/05/2018–14/05/2020
Gupta et al. (2021)	T-test and nonparametric test of Mann–Whitney	China, Japan, UK,USA, Germany and India	Stock indexes	01/01/2019–30/06/2020
Haroon and Rizvi (2020)	Exponential GARCH models and Ordinary Least Square Regressions	USA	Stock indexes and multisectoral analysis, differentiating travel and hotels	01/01/2020–30/04/2020
He et al. (2020)	Event study	China	Stock indexes and multisectoral analysis (no tourism)	03/06/2019–13/03/2020
Hung et al. (2021)	Event study	Vietnam	Stock market	02/01/2020–13/12/2020
Huo and Qiu (2020)	Event study	China	Multisectoral analysis (no tourism)	22/01/2020–03/03/2020
Jabeen et al. (2022)	Descriptive analysis	Usa, Germany, Australia, France, Europe, India, Japan, Pakistan and China	Stock indexes	01/01/2020–01/01/2020
Kusumahadi and Permana (2021)	Fundamental equation and TGARCH model	USA, Italy, Spain, Germany, China, France, UK, Canada, South Korea, Brazil, Australia, Indonesia, South Africa, Singapore and Morocco	Stock indexes	01/01/2019–30/06/2020
Liu et al. (2020)	Event study	Abu-Dhabi, France, Germany, USA, UK, Malaysia, Indonesia, Korea, Russia, Japan, Australia, Canada, Singapore, Thailand, China, Italy and India	Stock indexes	21/02/2019–20/03/2020
Liew (2020a)	Event study	China	Tourism	11/03/2019–14/04/2020
Liew (2020b)	Regression model	USA	Tourism and travel: touroperators	02/10/2019–27/03/2020
Mazur et al. (2021)	Descriptive analysis	USA	Stock indexes and multisectoral analysis, differentiating hospitality	01/03/2020–31/03/2020
Mdaghri et al. (2020)	Panel data regression	Middle East and North African (MENA) countries	Stock market	03/02/2020–20/05/2020
Nhamo et al. (2020)	Event study—descriptive analysis	Global	Tourism and Sports	19/02/2020–24/03/2020
Ofori-Boateng et al. (2021)	Event study	Ghana	Stock indexes	01/03/2020–01/01/2020
Ramelli and Wagner (2020)	Regression model	USA	Stock indexes and multisectoral analysis (no tourism)	31/12/2018–03/04/2020
Sharma and Nicolau (2020)	Autoregressive conditionaxl heteroskedasticity model	USA	Hotel, airline, cruise and rental car industries	01/09/2018–31/03/2020
Ramelli and Wagner (2020)	Event study and y least squares regression	USA	Stock indexes and multisectoral analysis (no tourism)	31/12/2018–03/04/2020
Schoenfeld (2020)	Regression model	USA	Stock indexes and comodities	01-2020–03-2020
Topaloglu et al. (2021)	Panel data regression	Turkey, Belgium, Germany, France, Italy, Spain, United Kingdom, United States, China and Netherland	Stock indexes and multisectoral analysis (no tourism)	17/03/2020–14/04/2020
Wu et al. (2021)	Event study and regression model	China	Tourism	25/03/2019–10/07/2020

*Source*: Authors’ own

As Table 1 indicates, there is abundant scientific literature on the effect of COVID-19 on the stock market worldwide, which can be seen in the behavior of its market indexes at the start of the pandemic (Schoenfeld 2020; Vila 2020): the Standard & Poor's 500 Index fell by 33.9%, the Euro Stoxx 50 and Russell 2000 fell by almost 40%, followed by the Dow Jones 30 Index, which plummeted to 32.3%; Ibex35 was among those that registered the greatest falls (39.4%), reaching its annual lows on March 16 and 18.

All the studies analyzed the initial effect of the pandemic; the most widely used methodologies include event studies (Ganie et al. 2022; He et al. 2020; Huo and Qiu 2020; Hung et al. 2021; Jabeen et al. 2022; Liu et al. 2020; Ofori-Boateng et al. 2021), panel data regression (Amin et al. 2021; Mdaghri et al. 2020; Topaloglu et al. 2021) and classic regression models (Ahmed 2020; Ahmed et al. 2021; Schoenfeld 2020), among others. These studies, especially those conducted in developed economies, reach a similar main conclusion: they confirm the significant negative effect of COVID-19 on the stock market.

Amin et al. (2021) analyze the stock market in developing countries, stating that “while a negative relationship was found between total number of cases and the stock market, a positive relationship was found between total number of deaths and the stock market” (Topaloglu et al. 2021: 37), where only recoveries from COVID-19 are considered the best predictor of stock market performance (Ahmed 2020).

The scientific literature (Table 1) is less extensive in terms of the stock market value of tourism-related companies (Al-Awadhi et al. 2020; Carter et al. 2022; Chai 2021; Gil-Alana and Poza 2022; Haroon and Rizvi 2020; Liew 2020a, b; Mazur et al. 2021; Nhamo et al. 2020; Sharma and Nicolau 2020; Wu et al. 2021). The authors reach conclusions similar to those indicated by studies conducted in the market generally. Of particular note is the significant adverse effect of COVID-19 on the tourism sector, with the stock prices of tourist companies falling significantly (Sharma and Nicolau 2020), exhibiting “extreme asymmetric volatility that negatively correlates with stock performance’ (Mazur et al. 2021:1). In fact, companies in the tourism sector and those related to its value chain displayed the poorest performance in global stock markets, losing up to 80% of their value over a period of two weeks (Nhamo et al. 2020).

As shown in Table 1, all studies conducted thus far have been conducted in the early stages of the pandemic. They coincide in their conclusions: the first outbreak of COVID-19 negatively affected stock prices in the tourism sector over and above other sectors (Gil-Alana and Poza 2022; Liew 2020b), with up to an 85% drop in tourism stock prices at the start of the outbreak, for three consecutive days (Liew 2020a).

The lockdown in Wuhan was a turning point in the behavior of the tourism sector stock market, with a faster decline observed in stock prices following this event in China (Al-Awadhi et al. 2020; Liew 2020b) and the United States (Carter et al. 2022). Additionally, the initial news of the COVID-19 outbreak and the measures taken to curb its spread slowed the stock market and resulted in a decline in the prices of tourism-related shares (Nhamo et al. 2020). Wu et al. (2021) confirmed that government interventions to appease the ASP were effective.

Stock prices during the COVID-19 pandemic have also been affected by the sentiment generated by news related to the coronavirus, which is associated with increased volatility in financial markets. This effect is more powerful in the sectors most affected by events driven by the pandemic, such as the hotel sector (Haroon and Rizvi 2020).

Therefore, after studying the conclusions of the scientific literature, the following hypotheses are proposed.

### H1

In the medium term, COVID-19 affects the tourism sector stock market negatively.

### H2

The negative effect of COVID-19 on the stock market is greater in the tourism sector than in the entire market.

From the entire corpus of literature on this subject, only Liew (2020b) and Sharma and Nicolau (2020) attempt to determine the effect of an increase in positive cases of COVID-19 on the stock market performance of tourist companies. There is a need to add the study carried out by Wu et al. (2021), which also analyzes the effect of the number of deaths from COVID-19, to this body of research. With the exception of Liew (2020b), who argue that variations in the number of positive cases have an insignificant effect on the tourism sector stock market, other studies confirm the inverse relationship between the health data of the pandemic and the stock market price listings of tourism companies. Based on the literature, the third hypothesis is as follows.

### H3

In the medium term, the number of deaths and positive cases of COVID-19 negatively affect the tourism stock price and are the best predictors.

Authors such as Ashraf (2020) argue that positive COVID-19 cases have a greater effect on the stock market than the number of deaths; therefore, the fourth hypothesis is formulated as follows:

### H4

The number of positive COVID-19 cases has a greater effect on tourism stock prices than the number of deaths.

However, at the start of the pandemic, during which all previous studies were conducted, the determination of the number of cases was not exact, and the real proportion of people infected was unknown (Ioannidis 2020). Bearing in mind the aforementioned limitation and considering that the number of deaths caused by COVID-19 during the study period was more realistic than the number of positive cases, the fifth research hypothesis examined here is as follows:

### H5

The number of deaths caused by COVID-19 is a better predictor of tourism stock prices than the number of positive cases.

The scientific literature establishes that cruise companies’ stock prices are the most affected subsector, followed by airlines and hotels, losing up to 80%, 67%, and 66% of their values, respectively (Carter et al. 2022; Nhamo et al. 2020; Sharma and Nicolau 2020). Carter et al. (2022) state that companies with the greatest financial leverage are the most heavily penalized.

## Data and methodology

To achieve the research outcomes established for this study, the data section below determines the explanatory and dependent variables that offer the best predictions. The methodology section further describes the applied statistical prediction model, which yields the results presented in the subsequent section.

### Data

The period spanned by the database used extends from the month in which the first cases and news related to the pandemic appeared until the vaccination process was implemented at a rate of 90%,^3^ and at least 5% of the population was fully immunized (Epdata 2021), from February 1, 2020, to March 31, 2021. This is a period of 14 months, which will allow the analysis to be conducted in the medium-term, going beyond the start of the pandemic, and covering the period of its highest incidence in Spain.

Different options were considered and tested for both the dependent and explanatory variables. The variables that yielded the best results were further chosen to develop the most reliable prediction model that would best define the relationship between the pandemic and the stock market performance of the tourism sector.

Stock price reflects the discounted value of a company's current and future performance. Significant changes in the price of shares can be attributed to special circumstances or events (Chen and Siems 2004), such as the COVID-19 pandemic.

For the dependent variable Y_t_, we analyze the stock market values of all companies in the tourism and traveler transportation sectors, which are listed on the Madrid Stock Exchange. According to the classification made by the latter, this sector comprises the following companies^4^: Amrest Holdings, S.E. (Amrest), Edreams Odigeo, S.A. (Edreams), Meliá Hotels International (Meliá), S.A., NH Hotel Group, S.A. (NH), Aena, S.M.E., S.A. (Aena), and International Consolidated Airlines Group (IAG). We should clarify that Amrest has been included, despite being in the restaurant subsector, because of the inevitable link that exists among the tourist companies. The potential dependent variables considered for modeling were as follows:

1. Companies’ daily stock prices.
2. Daily ASP is calculated using the approach to evaluating abnormal performance in a sector described by Brown and Warner (1985), applying the following formula for each day's values:
  1 $${ASP}_{jt}={C}_{jt}-\overline{{C }_{j}}$$
  where ASP_jt_ denotes the abnormal listed price of stock j at time t, C_jt_ denotes the actual observed listed price of stock j at time t, and C̅_j_ denotes the average of the daily stock prices listed on stock index j during the (−30,−11) estimation period. C̅_j_ is calculated as follows (Chen and Siems 2004):
  2 $${\overline{C} }_{t}=\frac{1}{20}\sum_{t=-30}^{-11}{C}_{jt}$$
3. Abnormal returns are calculated using the adjusted average return method and the following formula for the daily values:
  3 $$A{R}_{jt}={R}_{jt}-\overline{{R }_{j}}$$
  where AR_jt_ denotes the abnormal return of stock j at time t, R_jt_ denotes the actual observed performance of stock j at time t, and R̅_j_ denotes the average daily return of stock index j during the (−30,−11) estimation period. R̅ is calculated as follows (Chen and Siems 2004):
  4 $${\overline{R} }_{t}=\frac{1}{20}\sum_{t=-30}^{-11}{R}_{jt}$$

Regarding the values of series X_t_, four different variables for the behavior of the COVID-19 pandemic, obtained through the National Centre for Epidemiology (2021), were considered:

1. Number of daily deaths
2. Cumulative number of deaths
3. Number of new daily cases
4. Cumulative number of cases

**Fig. 1:**
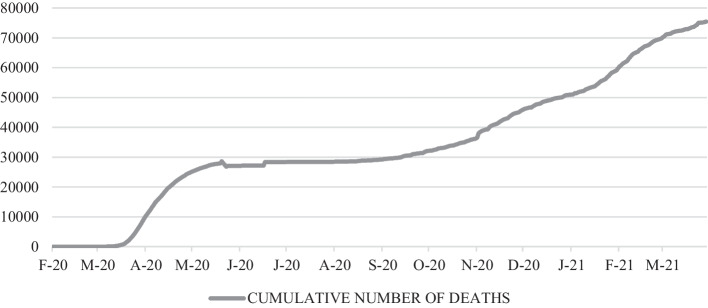
Cumulative number of deaths from COVID-19 (February/2020–April/2021). *Source*: National Centre for Epidemiology (2021)

**Fig. 2:**
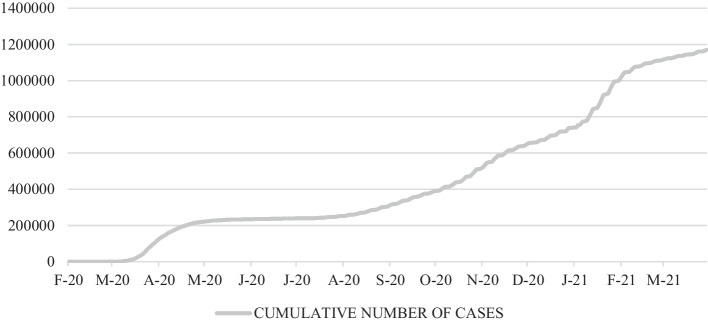
Cumulative number of cases of COVID-19 (February/2020–April/2021). *Source*: National Centre for Epidemiology (2021)

Lastly, the variables that yielded the best results were chosen. Regarding the dependent variable Y_t_, ASP, and explanatory variables X_t_, the cumulative number of deaths (Fig. 1) and the cumulative number of cases (Fig. 2) were selected.

### Methodology

First, a descriptive analysis was conducted in which the behavior of the ASP of the tourism sector and Ibex-35 are analyzed and compared, as a reflection of market evolution, as well as each of the companies in the sector, reporting average, maximum, and minimum values, and tying them in with circumstances and events related to the pandemic.

To conduct the statistical analysis, the classic regression model resolved by the least squares method was used as the starting point. However, this could not be applied to model dependency relationships between time series because the observations of a series depend on their past values. Therefore, the resulting residuals εt would be self-correlated, generating the following problems:

1. The estimation of β is no longer the best possible, as some information has been ignored in the calculations.
2. The t-test for the significance of the β coefficient shall be incorrect.
3. The Akaike information criterion (AIC) values of the adjusted models are no longer a good guide for the best prediction model.
4. The *p* values will be too small; therefore, some predictive variables will appear relevant when they are not, a situation referred to as spurious regression.
  5 $$Y_{t} = \alpha + \beta X_{t} + \epsilon_{t} ,\epsilon_{t} \sim N\left( {0;\sigma } \right)$$

In contrast, time series fit and prediction models, such as autoregressive integrated moving average (ARIMA) models (Stram and Wei 1986), make use of past observations but do not consider the inclusion of X_t_ series as explanatory covariables.

However, DRMs are a combination of the two methods described above (Bollerslev and Wooldridge 1992), which are well-known and widely used for fitting and prediction in almost all sciences (Ichev and Marinč 2018; Kaplanski and Levy 2010; Karafiath 1988; Lee and Jang 2007; Sharma and Nicolau 2020). In such regressions, errors can be self-correlated. DRMs allow us to capture the fact that the current value of a certain variable depends on its own past values and specifies independent variables (Mateu-Sbert et al. 2013). This study explores two time series, one of which can be used as an explanatory variable for the other. In this context, the regression model between series is usually used as if they were two statistical variables. However, it is modified so that errors are not self-correlated, which is why we chose this model.

The following equations show the exact formulation of a DRM, which has two associated error terms: η_t_ is associated with the regression of Y_t_ over X_t_, which is adjusted by an ARIMA(p,d,q) process; the final error ε_t_ is obtained from the ARIMA fit, which is required to yield independent and identically distributed values.

6 $$\begin{aligned} & Y_{t} = \alpha + \beta X_{t} + \eta_{t} , \\ & \eta_{t} \sim ARIMA\left( {p,d,q} \right) \\ & \eta_{t} \sim + \emptyset_{1} \eta_{t - 1} + \cdots + \emptyset_{p} \eta_{t - p} \\ & \quad + \theta_{1} \epsilon_{t - 1} + \cdots + \theta_{q} \epsilon_{t - q} + \epsilon_{t} \\ & \epsilon_{t } \sim N\left( {0;\sigma } \right) \\ \end{aligned}$$

The model described is estimated using the maximum likelihood method; the coefficients will take values that will ensure that the observed series is most likely to occur. One advantage of this method is that the estimated parameters have a known distribution; therefore, they can be associated with a hypothesis test to establish their influence on the model. Furthermore, the result is a parsimonious model, implying that the number of parameters considered is very small, which simplifies, facilitates, and refines the predictions.

Hence, the maximum likelihood estimation method ensures that the coefficients are unbiased estimations, asymptotically normal, and asymptotically efficient. Therefore, they are robust statistics; the *t*-test used to evaluate the significance associated with the coefficients is also robust.

We ruled out the use of other types of statistical models applied in previous studies (Table 1), such as data panel models (Al-Awadhi et al. 2020; Topaloglu et al. 2021). This is because, in these, the time variable is usually treated as a variable that stratifies the sample into a few categories, whereas in the research presented here, because it considers time series, there is a very fine partitioning of time. We also decided against using the event study method because the aim is to establish a predictive model of daily ASP, throughout 424 days (14 months), according to the health variables of COVID-19. The event study method is used to analyze the abnormal behavior of a financial asset in response to new information about a certain event, during an estimation period between 100 and 300 days, for studies with daily returns (Uguedo 2003), as has been done in previous studies that use this methodology (Liew 2020a; Liu et al. 2020; Ofori-Boateng et al. 2021).

For a DRM to be applied with full explanatory and predictive capacity, two restrictions must be met: stationarity of the series involved and non-correlation of residuals.

The series involved in a DRM must be stationary, meaning that both their mean and variance do not change over time. If they are not, the coefficients obtained are not consistent and/or significant estimates. To avoid this problem, one can convert non-stationary to stationary series using two methods: logarithmic transformation, which makes the dispersion more or less constant, or series differentiation, which helps eliminate the tendency of a series, seeking stationarity in the mean. These transformations were then applied to the variables. In practice, the stationarity of the series stock price is ensured using the Dickey-Fuller test (Said and Dickey 1984).

The second premise for the application of DRM is that the errors are white noise, indicating that εt behaves like a stochastic process taking random and independent values. In this study, the white noise condition was verified using the Ljung–Box test, which states in its null hypothesis that all autocorrelations in the series (up to a sufficiently high order) are null, which is equivalent to saying that the residuals are random and independent of each other (Ljung and Box 1978).

Bartlett’s test (Bartlett 1946) is an additional graphic test often used to support the Ljung-Box test (Bartlett 1946). This is a graphic representation test of autocorrelations ρ(k), orders 1, 2…, k. Theoretically, it can be proved that, in a series of white noise, such autocorrelations should be placed within the band “ − 2/T, 2/T”, with T being the size of the series.

Regarding goodness-of-fit, the most commonly used metric for evaluating the accuracy of a generic fit is the root-mean-square error (RMSE) (Chai and Draxler 2014). It is defined as the square root of the mean of the squared residuals and is expressed in the same units as the response variable used to compare different models.

7 $$RMSE=\sqrt{\frac{1}{n}\sum_{j=1}^{n}{\left({y}_{j}-{\widehat{y}}_{j}\right)}^{2}}$$

Therefore, the validation tests guarantee that the model performs well in the prediction of the results. The tests of normality and stationarity of residuals validate the hypotheses and guarantee that the resulting models are robust when the data fits well.

One way to select between models estimated with the maximum likelihood method is to use the RMSE; however, the higher the number of parameters, the better it will fit the data, although it will lose predictive capacity. In contrast, models with fewer parameters had the highest predictive capacity (principle of parsimony). The AIC statistic (Wagenmakers and Farrell 2004) considers the number of parameters to achieve a good compromise between fit and prediction. In this study, the lower AIC is used to select the definitive model from different correct explanatory models.

In summary, to choose the appropriate model, a massive screening of all possible dynamic models with explanatory variables and an error structure modeled by an ARIMA (p,d,q) process was performed, for which parameters p, d, and q were computationally varied over a wide range of values. From the resulting models, those that satisfied the condition that residuals ε and t were white noise were selected and verified using the Ljung–Box test. The stationarity of the series was confirmed using the Dickey-Fuller test. After filtering the models that proved suitable, the most parsimonious ones were selected, using the criterion of the minimum AIC, as a measure of the goodness of fit of the regressions obtained by the method of maximum likelihood.

## Results

### Descriptive analysis

Table 2 summarizes the results of the descriptive analysis.

**Table 2:** Descriptive analysis

	Subsector	Mean (%)	Median (%)	Minimum (%)	Maximum (%)
ASP Tourism	Tourism	− 0.40	− 1.13	− 47.81	50.34
ASP Ibex35	Market	− 0.34	0.08	− 35.76	19.44
ASP Amrest	Restaurant	− 1.14	− 1.07	− 52.32	53.20
ASP Edreams	Tour operator	2.41	2.62	− 63.47	62.41
ASP Meliá	Accommodation	0.49	− 1.28	− 58.6	62.57
ASP NH	Accommodation	− 0.40	− 3.85	− 52.75	59.76
ASP Aena	Air transport subsector	− 0.63	− 0.92	− 42.34	30.85
ASP AIG	Air transport subsector	− 3.11	− 1.44	− 66.56	64.10

*Source*: Authors’ own

**Fig. 3:**
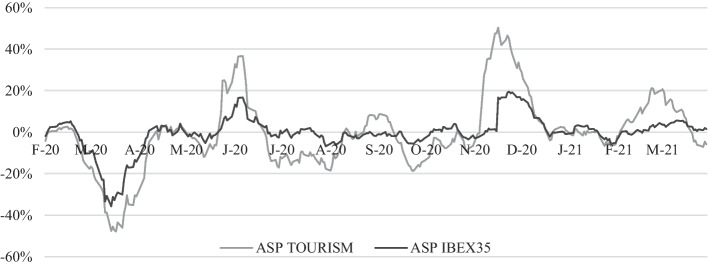
Abnormal stock prices of the tourism sector and the Ibex35 (February/2020–April/2021). *Source*: Madrid Stock Exchange (2021)

Throughout the pandemic, the ASP of shares on Ibex35, reflecting the behavior of the market, and those of the tourism sector listed on the Madrid Stock Exchange, are reflected in Fig. 1. As shown, especially in the case of tourism, these show negative or less positive values during the peak periods of COVID-19 in Spain: March and April, July and August, October and November 2020, and the end of January and February 2021. Figure 3 also shows that during the pandemic, ASP fluctuations were more pronounced in the tourism sector than in the market as a whole.

The effect of the pandemic on stock market values is noteworthy, and the ASP of the tourism sector showed values of − 48%, coinciding with the declaration of the State of Emergency on March 16, 2020, and the restriction on air and maritime transport in the Peninsula, the Canary Islands, and the Balearic Islands on March 20, 2020. The ASP of the Ibex35, on those days, accounted for − 35.76% (minimum value in the series) and − 29.07%, respectively. Therefore, it is safe to conclude that there are two moments to be highlighted in terms of their positive effect on the stock market: the lifting of strict national lockdown measures in May 2020, with the last extension of the state of emergency, which triggered stock prices, with a market ASP of 16.49% and 38.67% for the tourism sector. During this period, there was also an uptick in the ASP of the tourism sector on May 26, 2020 (24.85%), when it was announced that foreign tourists would no longer have to self-isolate.

Second, the highest value recorded for the tourism sector's ASP occurred on November 16, 2020. This is an upward trend favored by news of the effectiveness of COVID-19 vaccines on the 9th of that month when pharmaceutical companies Pfizer and BioNTech announced that the first phase of testing indicated that their COVID-19 vaccine was more than 90% effective (Ramón 2020). Additionally, on the 16th of that month, Moderna reported a preliminary efficacy of 94.5% for its vaccine (Ansede 2020).

**Fig. 4:**
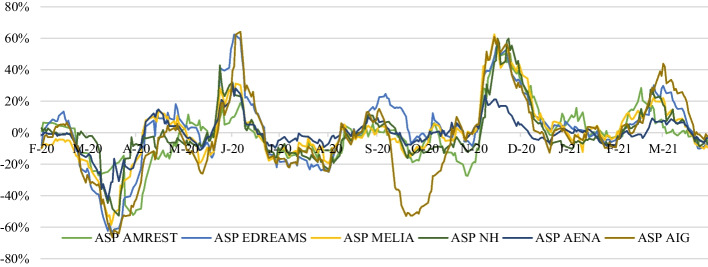
Abnormal stock prices of companies in the tourism sector (February/2020–April/2021). *Source*: Madrid Stock Exchange (2021)

The evolution of the ASP of companies in the tourism sector listed on the Madrid Stock Exchange is shown in Fig. 4. Generally, they all show similar behavior, more or less marked, with clear periods of upward and downward trends.

The company IAG of the passenger air transport subsector, with an average ASP of -3.11%, was the most volatile, with more pronounced maximum and minimum values than the rest of the companies. It should be noted that the ASP of September 2020 is the result of a process of capital increase, in which the listed price of subscription rights collapsed and dragged stock prices down with them (de la Quintana 2020).

The second most volatile company, as shown in Fig. 4, is Edreams (tour operator subsector), with an average ASP of 2.41%, indicating that it is more sensitive to its price increases than decreases.

The hotel subsector, for its part, has been the least volatile in the sector during the pandemic, with an average ASP closer to 0 than the rest (NH -0.40% and Meliá 0.49%).

### Statistical analysis

The results obtained are detailed below, after applying the methodology described in the third section to establish the relationships of dependency between the time series of ASP in the tourism sector (taken as explained variables Y_t_ and called ASP-tourism) and the series that defines the evolution of pandemic X_t_ considered explanatory variables; thus, we can demonstrate that there is a significant relationship of dependence between the two series, quantify the strength of this relationship, and establish a prediction model for ASP-tourism variables according to the evolution of the COVID-19 pandemic.

Tables 3 and 4 present the final results. Two DRMs that fulfill the hypotheses that ensure their correct functioning, with high predictive capacity, and in which the RMSE is very but not entirely similar, have been developed. In both cases, we had to apply logarithmic transformation to the predictor series X_t_ and use the series in differences of order d = 1 to ensure stationarity.

**Table 3:** Result of the dynamic regression model: explanatory variable “cumulative number of deaths”

Model Y_t_ = α + βX_t_ + η	Dickey–Fuller test	Estimation coefficients					AIC	RMSE	Ljung–Box test
			Estimate	Std. error	Z	*p* Value			
Y_t_ = ASP-tourism X_t_ = log_10_(deaths + 1) η_t_ ~ ARIMA(2,1,1)	DF = − 16.6 d = 1 lag = 0 *p* Value = 0.01	ar1	0.619	0.155	3.99	0.000**	660.4	0.72	Q* = 9.8 df = 6 *p* Value = 0.1
		ar2	0.152	0.068	2.22	0.026*			
		ma1	− 5.77	0,149	− 3.85	0.000**			
		β	− 3.725	0.860	− 4.33	0.000**			

*Source*: Authors’ own

*p* Value: *p* < 0.1; *p < 0.05; **p < 0.0

**Table 4:** Result of the dynamic regression model: explanatory variable “cumulative number of cases”

Model Y_t_ = α + βX_t_ + η	Dickey–Fuller test	Estimation coefficients					AIC	RMSE	Ljung–Box test
			Estimate	Std. error	Z	*p* Value			
Y_t_ = ASP-tourism X_t_ = log_10_(Cases + 1) η_t_ ~ ARIMA(1,1,1)	DF = − 16.6 d = 1 lag = 0 *p* Value = 0.01	ar1	0.976	0.025	38.2	0.000**	615.1	0.73	Q* = 9.6 df = 7 *p* Value = 0.2
		ma1	− 0.788	0.049	− 15.8	0.000**			
		β	− 16.448	3.658	− 4.5	0.000**			

*Source*: Authors’ own

*p* Value: p < 0.1; *p < 0.05; **p < 0.0

In the prediction model of the ASP of the tourism sector based on the total number of deaths caused by COVID-19 (Table 3), all the coefficients obtained were significant, particularly the β coefficients associated with the COVID-19 explanatory series (cumulative deaths), which were significant at 99%, evidencing the effect of the pandemic on the ASP of the tourism sector. The negative sign of β implies that there is an inverse relationship between the total number of accumulated deaths caused by COVID-19 and the ASP in the tourism sector. A one-unit increase in the number of deaths drives a fall in the ASP of − 4.33 units (Table 3, coefficient Z). Therefore, the negative effect of deaths caused by COVID-19 on the ASP of the tourism sector is certified by this model.

Regarding the prediction model of the ASP of the tourism sector based on the total number of positive cases of COVID-19 (Table 4), the β coefficients were also significant at 99%, demonstrating the effect of COVID-19 on the ASP of the tourism sector. The negative sign is consistent with the effect of declining listed prices when the effect of the pandemic increases. Therefore, the value of Z − 4.55 is interpreted as the change in units of the variable ASP-tourism that has grown in one unit of the variable cumulative number of cases. Once again, the negative effect of COVID-19 on the tourism sector stock market is confirmed, although this time, through the number of positive cases of infection, whose variation generates slightly greater uncertainty in the markets in view of the Z value.

Regarding the complexity of the models, the most parsimonious (simplest) model is the dependent model of the number of positive COVID-19 cases, which is a point in its favor. However, regarding the residual error, the model based on the cumulative number of deaths has an RMSE of 0.72, which is slightly below the RMSE error of the number of diagnosed cases (0.73). Therefore, it can be considered a better predictor, albeit with very little difference.

The Dickey-Fuller test states that a series with no lag (lag = 0) and differentiated d times is not stationary; this is the case in both models (Tables 3, 4). Additionally, ideally, the p-value of the test should be less than 0.05; in this case, for both explanatory models of ASP-tourism according to the cumulative number of deaths and the cumulative number of cases, this test returns a value of 0.01, which is below the appropriate value; therefore, stationarity is established.

The *p* values resulting from applying the Ljung–Box test were, in all cases, greater than 0.05. Therefore, the hypothesis that the residual variables are white noise holds. Moreover, both explanatory models of ASP-tourism based on the cumulative number of deaths and the cumulative number of cases yield a value of 0.1 (Table 3) and 0.2 (Table 4), respectively, above the appropriate value.

**Fig. 5:**
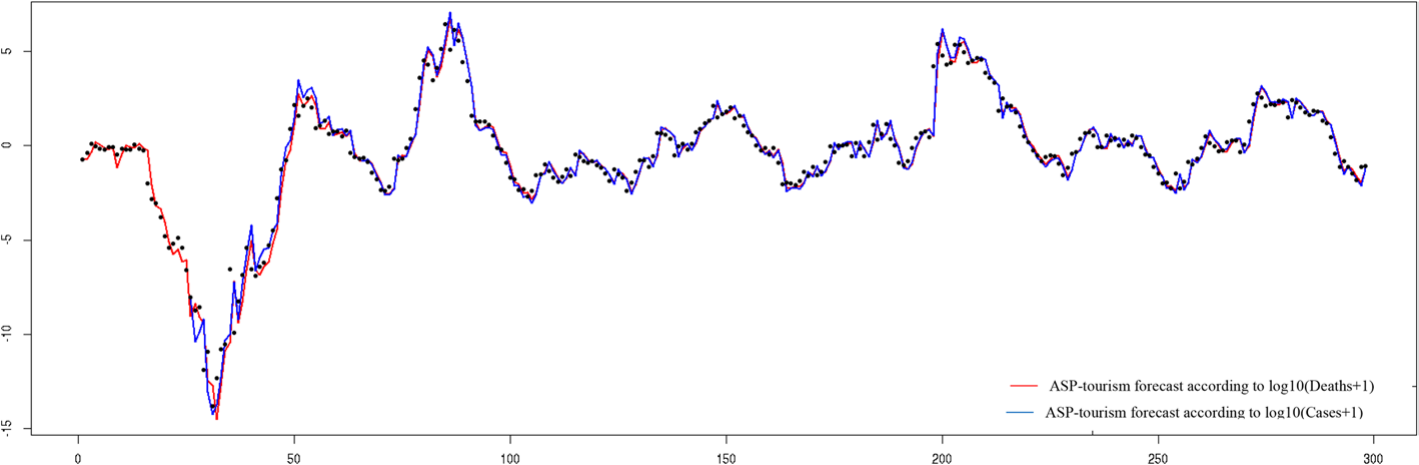
Fit of the tourism industry's abnormal stock price according to the confidence band of the applied model. *Source*: Authors’ own

Figure 5 shows the fit behavior and how the tourism sector's ASP moves within a 95% confidence band for the adjusted value, based on the explanatory variables of cumulative COVID-19 deaths and cases.

**Fig. 6:**
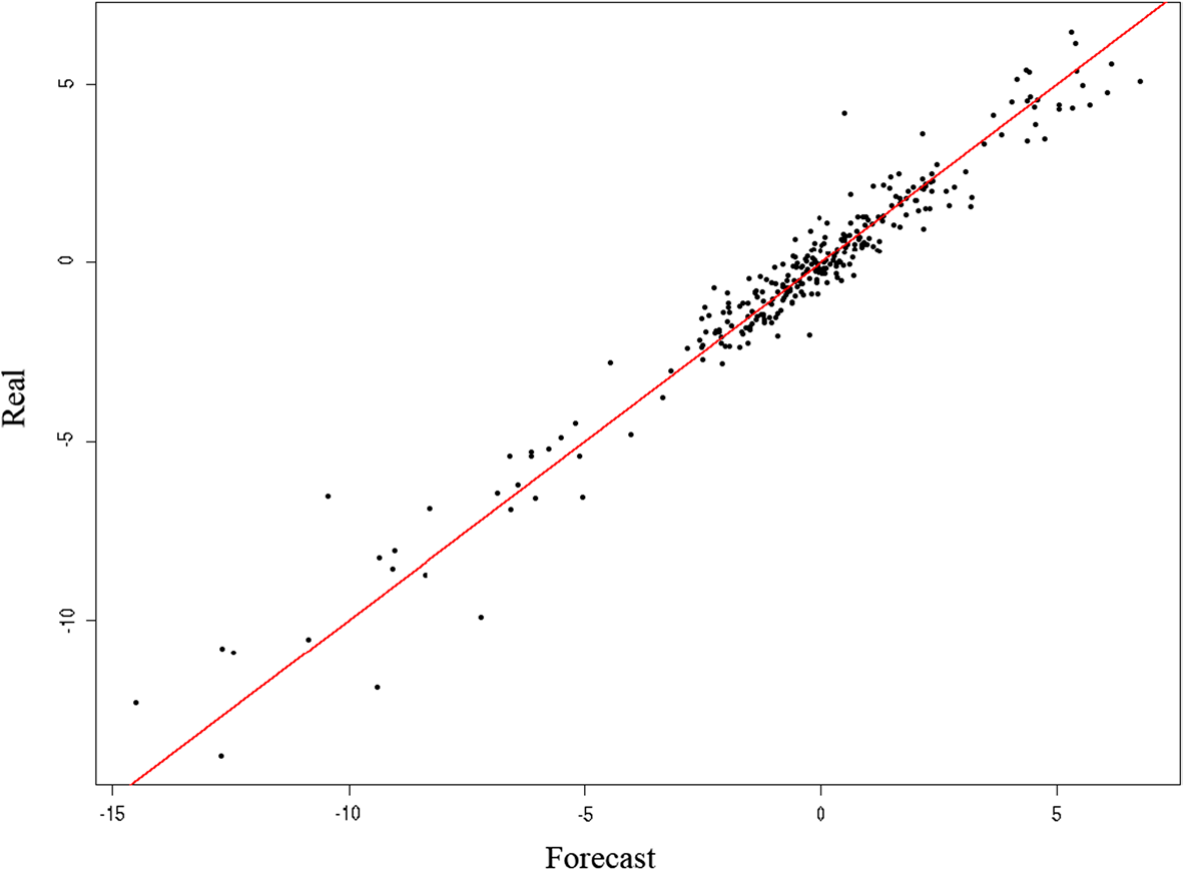
Goodness of fit of the model designed, described by the covariant of the series cumulative number COVID deaths. *Source*: Authors’ own

**Fig. 7:**
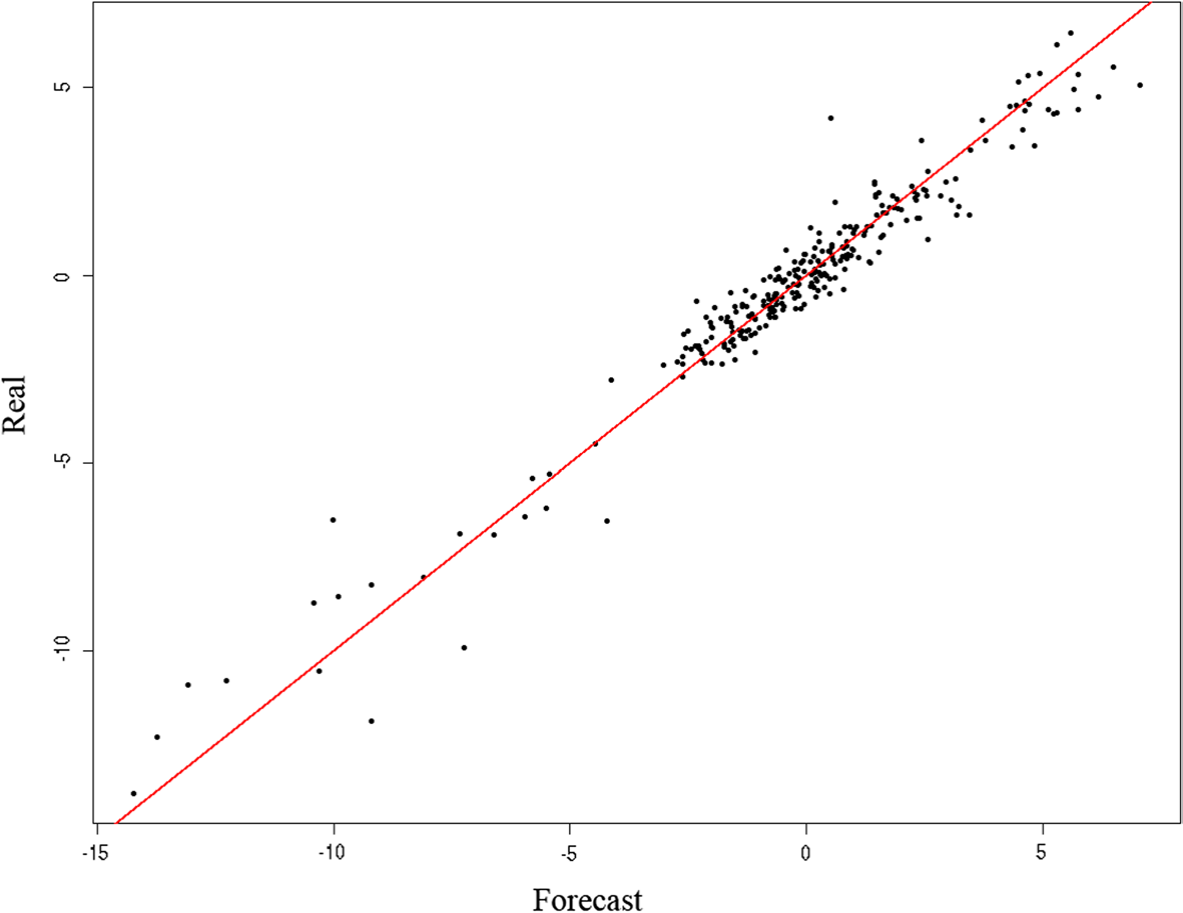
Goodness of fit of the model designed, described by the covariant of the series cumulative number of COVID-19 cases. *Source*: Authors’ own

Additionally, the goodness of fit of the designed models is confirmed in Figs. 6 and 7, showing that the actual values of the ASP-tourism series, represented in the cloud of points, fit fairly well with the values predicted by the models dependent on the COVID-19 variables. Investors, therefore, can use these models when making decisions to reduce their risk in epidemic situations similar to COVID-19, as the predictive capacity of the explanatory variables is confirmed once again.

**Fig. 8:**
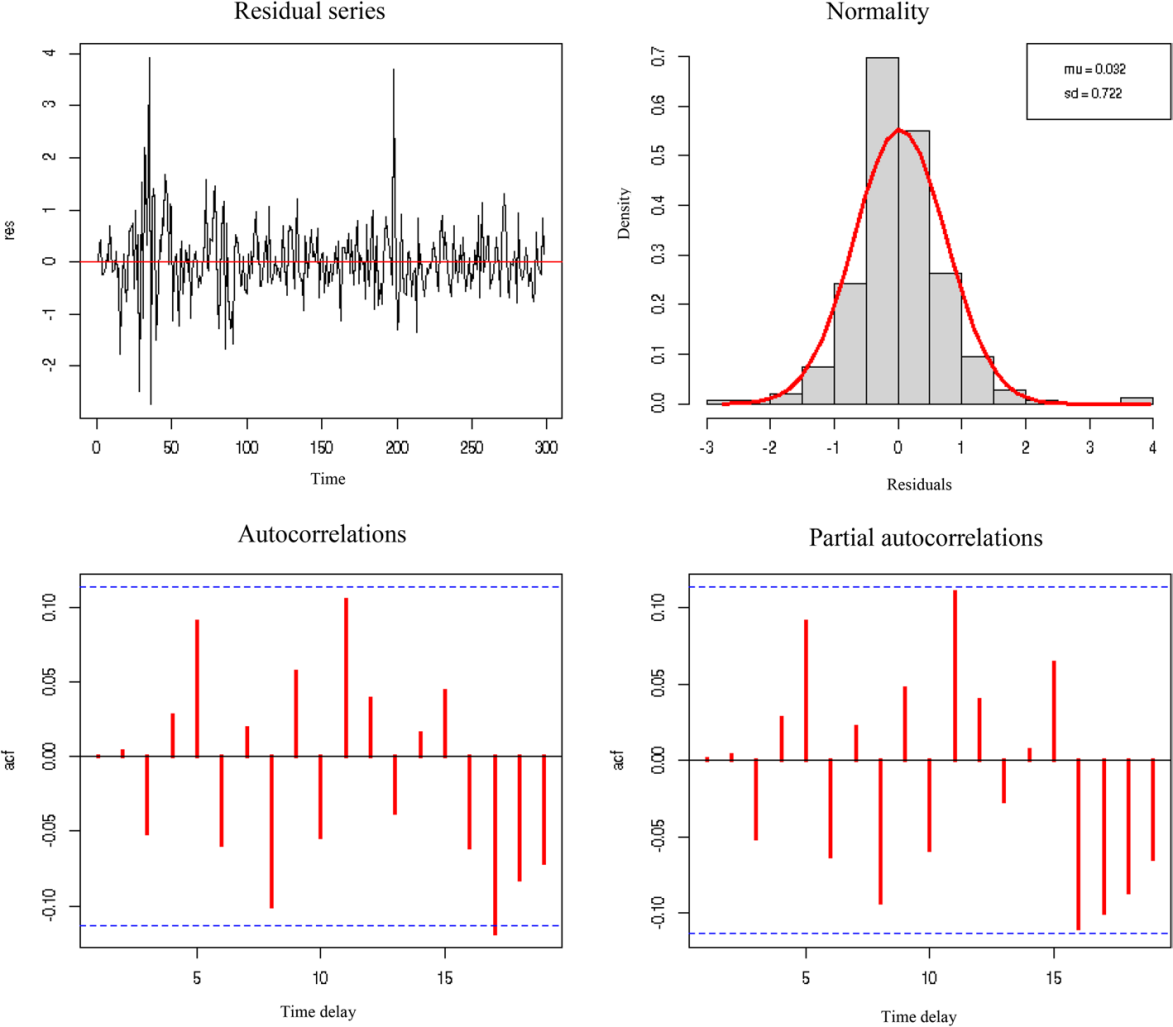
Residual series, normality, and autocorrelations of the abnormal stock prices in the tourist sector against the variable cumulative number of COVID-19 deaths. *Source*: Authors’ own

**Fig. 9:**
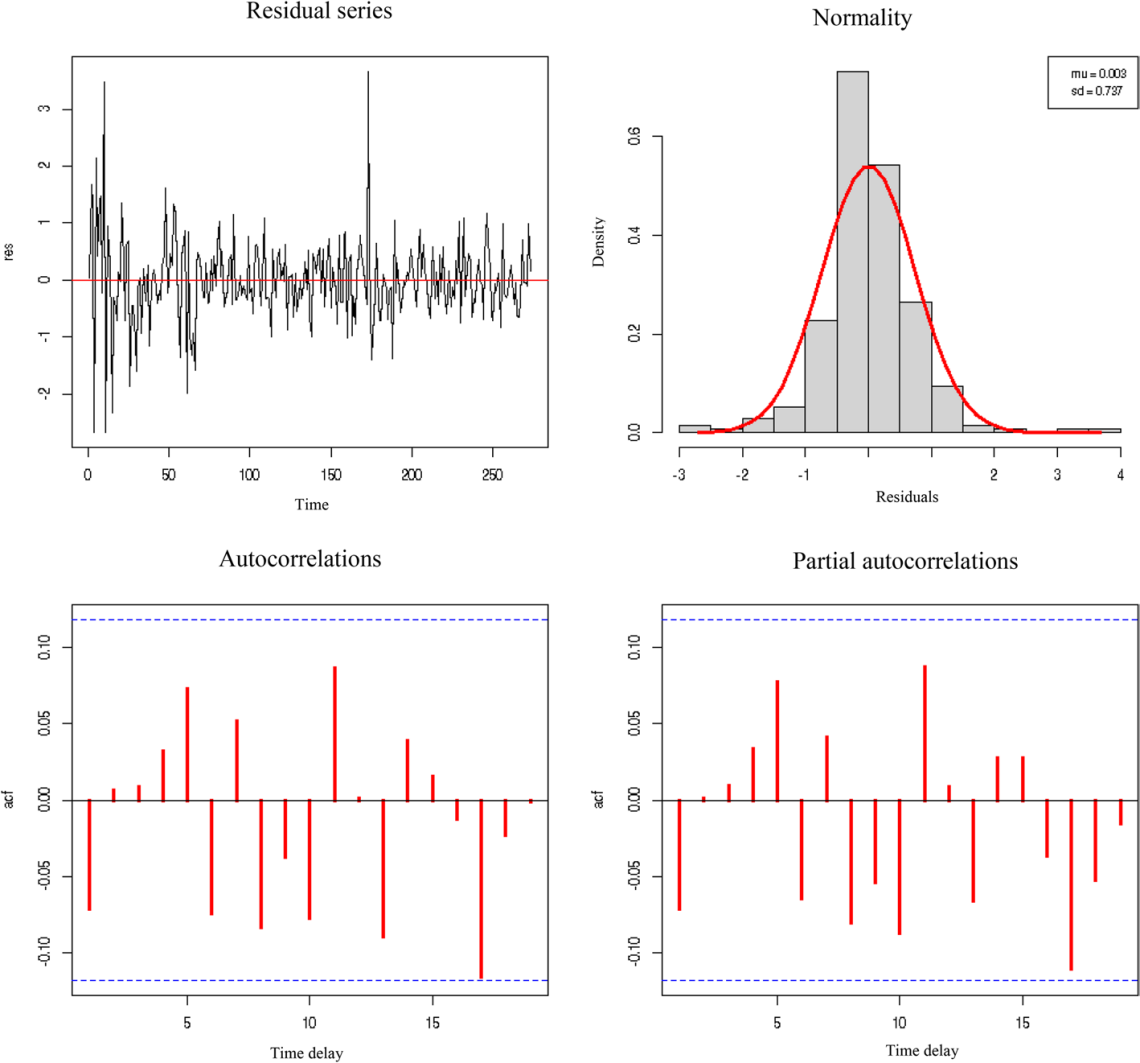
Residual series, normality, and autocorrelations of abnormal stock prices in the tourist sector against the variable cumulative number of COVID-19 cases. *Source*: Authors’ own

Lastly, Figs. 8 and 9 show the graphical verification of the behavior of the epsilon residuals, which must act as white noise without correlation or dependence, as is the case here.

Evidently, in both cases, the normal autocorrelations concerning “n” days before and partial autocorrelations concerning − 2 days of the residuals are not significant, as they are placed within the Bartlett bands. Therefore, the histogram can be approximated sufficiently well through normal distribution. Moreover, the evolution of residuals over time does not show growth in the mean or amplitude.

## Discussion

As the scientific literature advances (Carter et al. 2022; Liew 2020b; Mazur et al. 2021; Wu et al. 2021), COVID-19 has fully affected the tourism sector stock market, interacting negatively with stock market returns (Al-Awadhi et al. 2020) and which has been confirmed by the results of this research. Sharma and Nicolau (2020), Bartik et al. (2020), and Nhamo et al. (2020) stressed that, at the beginning of the pandemic, the decline in the tourism sector's stock market prices reflected the severity of this pandemic, when governments adopted the most restrictive measures in a bid to contain the pandemic, which is among the main reasons the stock market reacted strongly to COVID-19 (Baker et al. 2020).

These statements are confirmed by this study, as the greatest decline in the tourism sector ASP was noted in March 2020. However, this effect was also ratified over the course of the 14 months analyzed. This corrects the statements made by Verma (2021), who argued that the markets only reacted negatively to COVID-19 in the short term, or by Gil-Alana and Poza (2022), who contended that this negative effect disappears over time.

Our work confirms that the tourism sector was affected more than the market overall in both the short and medium terms and at the onset of the pandemic (Nhamo et al. 2020). A similar conclusion was reached by Liew (2020b) and Chen et al. (2007) regarding the SARS-2003 outbreak and by Ichev and Marinč (2018) regarding the stock exchange's response to the Ebola crisis.

The tourism sector ASP showed negative or less positive values than the market during the periods when there were peaks of COVID-19 in Spain. Al-Awadhi et al. (2020) in China, Sharma and Nicolau (2020) in the United States, and Nhamo et al. (2020) globally, show that, in the first stage of the pandemic, the stock market behavior of the tourism market was significantly worse than that of the market overall. This study confirms that the ASP of the tourism sector reacted more significantly than the market over the course of the pandemic and, in the short term, with more pronounced variations, demonstrating the sensitivity of the sector to COVID-19.

The cumulative numbers of cases and deaths are the best predictors of the evolution of stock prices in the tourism sector during the global COVID-19 pandemic. Moreover, there is an inverse relationship between them (Sharma and Nicolau 2020; Wu et al. 2021). This demonstrates that the significant positive relationship observed between the total number of deaths from COVID-19 and the stock market, confirmed by Topaloglu et al. (2021) in March 2020, is not noted in the tourism sector over the course of the pandemic. Hence, the ASP of tourist companies is more volatile in the face of fluctuations in the cumulative numbers of COVID-19 cases and deaths, as reflected by Mazur et al. (2021). Therefore, these variables have a high predictive capacity for the ASP of the tourism sector.

Each variation in the number of positive cases has a slightly greater effect on the tourism sector’s ASP than the number of deaths because the estimation coefficient Z for β is higher for the first explanatory variable than for the second (Tables 3, 4), coinciding with the conclusions reached by Ashraf (2020). Despite what Liew (2020b) indicated for the United States, where the variation of the new daily coronavirus cases in the world has a negligible effect on the stock prices of the tourism sector.

Lastly, the number of deaths caused by COVID-19 offers a better prediction than the number of positive cases because the model based on the first explanatory variable has an RMSE slightly below that of the second one (Tables 3, 4). This is justifiable as, at the start of the pandemic, the number of people infected was not an exact figure, given the tests conducted (Ioannidis 2020).

## Conclusions

The COVID-19 pandemic has shaken the global and Spanish economies and the tourism sector. Based on the stock market prices of Spanish tourism companies, this research has achieved its intended goals and has validated the hypotheses established, filling a gap in the scientific literature.

This study has confirmed the negative effect of COVID-19 on the tourism sector stock market in the medium term (H1) and that the ASP showed maximum and minimum peaks throughout the pandemic and in the first few weeks, which are more marked than those reflected by the market itself, which track the evolution of the pandemic (H2).

Two prediction models for the ASP of tourist companies using the DRM have been developed. In view of the results obtained, it is safe to conclude that the number of deaths and positive cases of COVID-19 negatively affect the tourism sector stock price and that both are good predictors (H3). The number of cases has a slightly higher effect on stock market prices (H4), although the cumulative number of deaths is the best explanatory variable in terms of predicting the ASP behavior of the tourism sector (H5).

The identification of the key predictors of stock prices and the creation of models to predict their effects on the stock market allow for financial decision-making (Kou et al. 2021). Therefore, the results of this research are fundamental to policymakers, governments, and investors, as they help decision-makers manage possible future outbreaks of this pandemic or similar epidemiological situations with the least possible effect.

The models examined here allow us to know and anticipate changes in the stock market prices of companies and can be used as a tool to objectively decipher the effect of pandemics, such as COVID-19. Governments and policymakers should focus their efforts on reducing investor uncertainty, stimulating the liquidity of the stock market, and supporting the tourism market rationally (Mdaghri et al. 2020).

Behavioral finance studies (Ichev and Marinč 2018) show that investors’ decisions are influenced by their feelings and, therefore, may affect the price of shares; therefore, the negative effect on stock prices could be lower, minimizing uncertainty about shares and maintaining investors’ confidence, preventing drastic and widespread variations over time (Chen et al. 2009). Investors are naturally risk-averse, especially in such situations. Therefore, they should pay close attention to the number of deaths and positive cases to guide their decisions and apply caution in long-term decisions. We recommend diversifying investment portfolios with assets into more secure and less volatile sectors.

One of this study’s limitations is its geographical scope, which focuses solely on Spain. Furthermore, it has not considered other control variables, such as events and news stories related to COVID-19 or the characteristics of the environment. Regarding the methodology, we use DRM and ARIMA modeling following a linear data structure assumption. Furthermore, statistical data on health during pandemics are nonlinear by nature (but there is a linear relationship between the series considered). Another limitation is the possibility of applying another methodology, such as panel models.

The proposed future lines of research include analyzing the effect of these explanatory variables on the ASP of each tourism subsector to better understand the effects of the pandemic. It would also be useful to expand the geographical scope of this study to other countries and compare the results. Establishing control variables such as events and news stories related to COVID-19, liquidity risk, return, or profitability, which would further hone the balance between fit and prediction, is another possible future strand of research. It would be interesting to evaluate the effect of the pandemic, specifically, through "the negative coefficient of skewness" and the "Down-to-Up Volatility" of firm-specific returns, which gave rise to excellent results in previous studies (Wen et al. 2019).

Finally, it would be beneficial to apply other methodologies, such as multifactor CAMP, panel models, or event studies, to compare the conclusions reached here and even assume the nonlinear character of the series used and make the results more robust.

## Data Availability

The datasets analysed during the current study are available in Madrid Stock Exchange (https://www.bolsademadrid.es) and National Centre for Epidemiology (https://cnecovid.isciii.es/covid19/).

## Abbreviations

Aena: Aena, S.M.E
AIC: Akaike information index
Amrest: Amrest Holdings, S.E.
ASP: Abnormal stock price
Edreams: Edreams Odigeo, S.A.
DRM: Dynamic regression model
GDP: Gross domestic product
IAG: International Consolidat. Airlines Group
Meliá: Meliá Hotels International S.A.
NH: NH Hotel Group, S.A.
RMSE: Root-mean-square error

## Footnotes

1 The Ibex 35 is a market capitalization weighted index made up of the thirty-five most liquid securities in the continuous market of the Spanish stock market (Sala and Yzaguirre 2003). The Ibex 35 is used as a benchmark or reference index because it is the one that most completely represents the Spanish stock market (Valdemar and Martinez 2013).

2 It is understood that the short-term horizon implies periods below one year (Cassola and Morana 2004), even less, between a week and a month (Jegadeesh 1990). Therefore, the following time frame is considered as medium term.

3 Number of doses administered over those received.

4 The company TR Hotel Jardín del Mar, S.A. is part of this group of companies; however, it did not provide sufficient data to be considered in this study.
